# Gamma-Glutamyl Transferase and Male Reproductive Hormone Levels in Men with Type 2 Diabetes: A Cross-Sectional Study

**DOI:** 10.2174/0118715303392699251016073511

**Published:** 2026-01-12

**Authors:** Yujie Feng, Hui Zhao, Runqi Zhang, Qingbo Guan, Zhenhua Tian

**Affiliations:** 1 Multi-disciplinary Treatment Department, Shandong Provincial Hospital Affiliated to Shandong First Medical University, Jinan, Shandong, 250021, China;; 2 Key Laboratory of Endocrine Glucose & Lipids Metabolism and Brain Aging, Department of Endocrinology, Ministry of Education, Shandong Provincial Hospital, Shandong University of Traditional Chinese Medicine, Jinan, Shandong, 250021, China;; 3 Key Laboratory of Endocrine Glucose & Lipids Metabolism and Brain Aging, Department of Endocrinology, Ministry of Education, Shandong Provincial Hospital Affiliated to Shandong First Medical University, Jinan, Shandong, 250021, China;; 4 Division of Geriatrics, Shandong Provincial Hospital Affiliated to Shandong First Medical University, Jinan, Shandong 250021, China

**Keywords:** Gamma-glutamyl transferase, male reproductive hormones, testosterone, sex hormone-binding globulin, type 2 diabetes, metabolic disorders

## Abstract

**Introduction:**

Although Gamma-Glutamyl Transferase (GGT) has been implicated in metabolic disorders, its association with male reproductive hormones in type 2 diabetes mellitus (T2DM) remains unclear.

**Methods:**

In this cross-sectional study, 971 men with T2DM were recruited from Shandong Provincial Hospital. Serum GGT levels were measured as the primary exposure variable, while male reproductive hormones—including total testosterone (TT), follicle-stimulating hormone (FSH), luteinizing hormone (LH), and sex hormone-binding globulin (SHBG)—served as outcome variables. Multivariable linear regression, threshold effect models, and subgroup analyses were used to assess associations, adjusting for age, BMI, glycemic control, and other confounders.

**Results:**

The mean age of participants was 56.37 ± 12.49 years. After full adjustment, men in the highest GGT tertile exhibited a 0.44 ng/mL reduction in TT levels compared to the lowest tertile (*p* < 0.01). A nonlinear relationship was identified, with a saturation effect at GGT = 118 IU/L. Below this threshold, each 10-IU/L increase in GGT corresponded to a 0.13 ng/mL decrease in TT (95% CI: -0.21 to -0.05). Unadjusted inverse associations with SHBG (β = -14.87, 95% CI: -26.47 to -3.28) and LH (β = -1.34, 95% CI: -2.14 to -0.54) became nonsignificant after adjustment (*p*>0.05). No FSH associations were detected.

**Discussion:**

Our findings reveal a novel dose-dependent relationship between GGT and TT in T2DM, with effects plateauing beyond 118 IU/L. This threshold may reflect the point where oxidative stress overwhelms gonadal compensatory mechanisms.

**Conclusion:**

The nonlinear relationship between GGT and TT suggests GGT may help identify androgen deficiency risk in T2DM though SHBG/LH links appear confounder-dependent. Monitoring GGT could aid T2DM male health management.

## 1 INTRODUTION

The prevalence of diseases such as diabetes mellitus (DM) and hypogonadism is increasing rapidly in aging communities worldwide. Low testosterone levels have been observed in 25-40% of type 2 DM (T2DM) patients [1-3]. Low testosterone levels are associated with symptoms such as fatigue, low libido, and erectile dysfunction [3]. However, recent studies have shown that in addition to the effects related to sexual health, subnormal testosterone levels have other pathophysiological effects, including a decrease in hematocrit levels, a reduction in bone mineral density, an increase in the risk of coronary artery disease [4], and an increase in the occurrence of cardiovascular events [5] and all- cause mortality [6-8]. Patients with hypogonadism have been reported to have increased oxidative stress, characterized by an increase in total oxidant status and a decrease in total antioxidant capacity and thiol levels. These parameters improved after 6 months of hypogonadism treatment [9]. In diabetes patients, intermittent hyperglycemia triggers oxidative stress and endothelial dysfunction, which leads to the development of microvascular and macrovascular diabetic complications [10, 11].

As a surface enzyme that catalyzes the transfer of gamma-glutamyl functional groups from glutathione to other acceptors to regulate the redox status, gamma-glutamyl transferase (GGT) is commonly used as a marker of liver dysfunction. It has also been suggested as an early marker of oxidative stress and inflammation [12]. Elevated GGT activity has been reported to be associated with diabetes, metabolic syndrome, and cardiovascular risk factors [13-15]. However, observational studies on the association of GGT with male reproductive hormones are scarce, and the association between GGT levels and sex hormone concentrations is still inconclusive. A cross-sectional study including 245 male partners of pregnant women found that GGT levels were not related to the levels of reproductive hormones [16]. Another study reported that total testosterone (TT), sex hormone-binding globulin (SHBG), and dehydroepiandrosterone sulfate (DHEAS) levels were associated with GGT levels in age-adjusted (but not in multivariable) ordinary least square regression models. Still, longitudinal analyses showed that baseline TT, SHBG, free testosterone, and DHEAS concentrations were also inversely associated with changes in GGT levels after multivariable adjustment [17]. Only one study involving men with diabetes found that the correlations between SHBG and GGT levels were prominent after adjusting for age and sex [18]. Despite these studies, data on the associations between GGT levels and levels of other sex hormones in men with diabetes remain inconclusive. Therefore, this study aimed to investigate whether GGT levels were independently related to male reproductive hormone levels in men with T2DM.

## 2 MATERIALS AND METHODS

### 2.1 Study Design and Participants

We conducted a cross-sectional study to explore the relationship between GGT and male reproductive hormones in Chinese male patients with T2DM. The target independent variable was the GGT level at baseline. The dependent variables were the levels of male reproductive hormones, including TT, SHBG, follicle-stimulating hormone (FSH), and luteinizing hormone (LH). Participants were non-selectively and consecutively enrolled from the Department of Endocrinology at Shandong Provincial Hospital in Jinan, Shandong, China. We did not include identifiable participant data to safeguard patient privacy. Data were obtained from the hospital’s electronic medical record system. Due to the nature of this cross-sectional study, informed consent from participants was not required. The hospital institutional review board approved this study (No. SWYX2025-335).

**Fig. (1):**
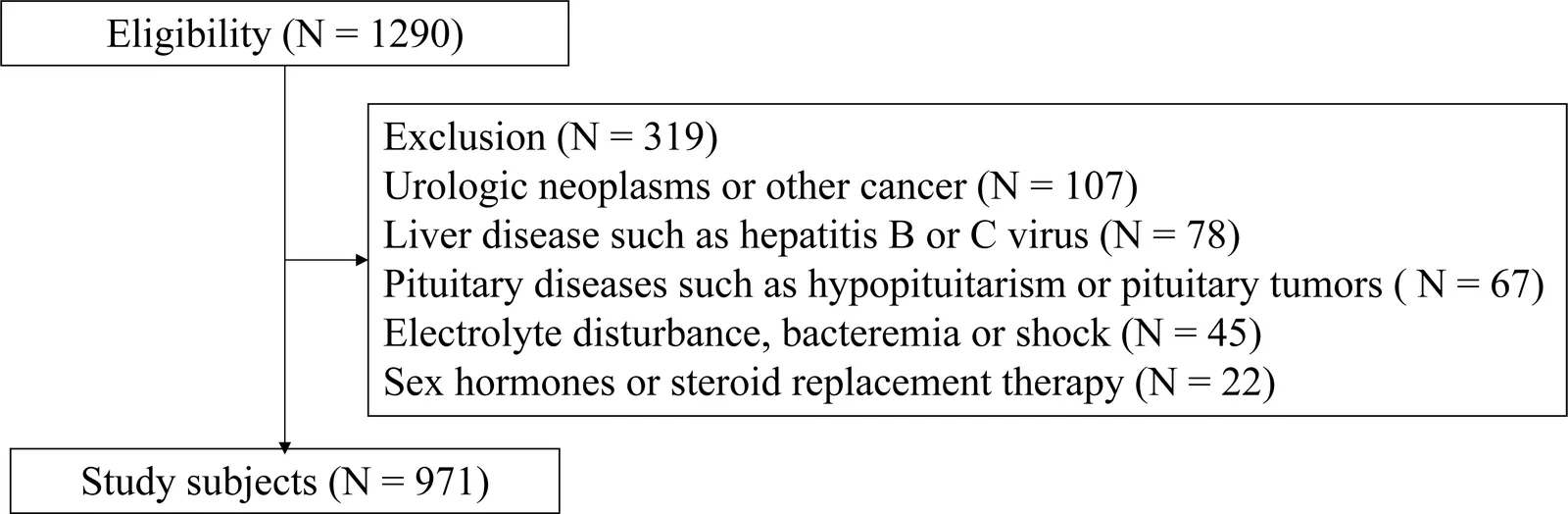
Flow diagram of the screening and enrollment processes. Among the 1,290 patients who available laboratory results, 971 were involved in the final analyses.

We reviewed the medical records of patients with T2DM who were hospitalized in Shandong Provincial Hospital between December 2016 and June 2020. We included 1,290 men aged ≥ 18 years who had available laboratory results for the levels of GGT and male reproductive hormones, including TT, FSH, LH, and SHBG. The exclusion criteria included the following: participants with a history of urologic neoplasms or other cancers (n = 107); who had known hepatitis B or C virus-related liver disease (n = 78); who had known pituitary diseases such as hypopituitarism or pituitary tumors (n = 67); who had electrolyte disturbance, bacteremia, or shock (n = 45); and who had received treatment with male reproductive hormones or steroid replacement therapy (n = 22) in the past week. Finally, 971 participants with diabetes were involved in the final analyses (Fig. **1**).

### 2.2 Measurements

The covariates used in this study can be divided into the following categories: (1) demographic data; (2) our prior work; (3) other studies examining the factors associated with male reproductive hormones; and (4) our clinical experiences. The following variables were used to construct multivariable adjusted models: (1) continuous variables: age, Body Mass Index (BMI), glycated hemoglobin (HbA1c), insulin (INS), c-peptide (CP), asting plasma glucose (FPG), estimated glomerular filtration rate (eGFR), GGT, alanine aminotransferase (ALT), aspertate aminotransferase (AST), triglyceride (TG), total cholesterol (TC), high-density lipoprotein cholesterol (HDL), and low-density lipoprotein cholesterol (LDL), Uric acid (UA) levels (obtained at baseline); and (2) categorical variables: presence of hypertension, smoking status, and alcohol consumption.

Diabetes was defined as a previous diagnosis made by a healthcare professional, as FPG level ≥ 7.0 mmol/L, or as a 2-h PG level of ≥ 11.1 mmol/L. Hypertension was indicated by a systolic blood pressure of ≥ 140 mmHg, a diastolic blood pressure of ≥ 90 mmHg, or a previous diagnosis of hypertension made by a physician.

The intra-assay and inter-assay coefficients of variation were always below 5% for all of the above parameters. Blood samples were obtained between 6:00 and 9:00 a.m., after the participants had fasted for at least 8 h. Blood was immediately refrigerated after phlebotomy, centrifuged within 2 h, and the serum was then aliquoted and frozen in the central laboratory. Electrochemiluminescence immunoassays (Roche Cobas E801, Basel, Switzerland) were used to measure TT, SHBG, FSH, LH, insulin, and CP levels. FPG, UA, serum creatinine, TG, TC, HDL, and LDL levels were measured with an Olympus AU5400 system (Tokyo, Japan). HbA1c levels were measured by high-performance liquid chromatography (G8, TOSOH, Japan).

### 2.3 Statistical Analysis

Data are presented as the mean ± standard deviation (SD) for normally distributed continuous variables, the median (*Q*1–*Q*3) for skewed continuous variables, and the frequency (percentage) for categorical variables. For the analysis of baseline characteristics, statistical differences among tertiles of GGT levels were tested with the χ^2^ test for categorical variables, one-way analysis of variance for normally distributed continuous variables, and the Kruskal–Wallis H test for skewed continuous variables. β values and 95% confidence intervals (CIs) were calculated for serum GGT levels using univariate and multivariate binary logistic regression. Both unadjusted and multivariate-adjusted models were used. In the adjusted model I, age and BMI were included. In adjusted model II, model I covariates were included, and the regression coefficient of each covariable to Y is *p* < 0.1, including CP, ALT, HbA1c, INS, LDL, TG, HDL, UA levels, smoking status, and alcohol consumption. To assess confounding factors, we input the covariates for regression into the basic model or eliminated the covariates in the complete model one by one and compared the regression coefficients. The trend tests were performed by inputting the median value of each GGT tertile as a continuous variable in the linear regression models.

A generalized additive model was used to evaluate the nonlinear relationship between GGT levels and male reproductive hormone levels. According to the smoothing curve, we further established a two-piecewise linear regression model to identify the threshold effect, with adjustment for potential confounders. The threshold GGT level was determined using a recurrence method, which involved selecting the turning point along a predetermined interval that produced a maximum likelihood model. A log-likelihood ratio test was used to compare the two-piecewise linear regression model with the one-line linear model. Subgroup analyses were performed by age and hypertension *via* stratified regression. Interaction analysis across subgroups was performed using the likelihood ratio test.

All analyses were performed using R Statistical Software (Version 4.2.2, http://www.R-project.org, The R Foundation) and Free Statistics analysis platform (Version 2.2, Beijing, China). A two-sided *p* value <0.05 was considered to be statistically significant.

## 3 RESULTS

### 3.1 Baseline Characteristics of the Study Participants by Categories of Serum GGT Levels

**Fig. (2):**
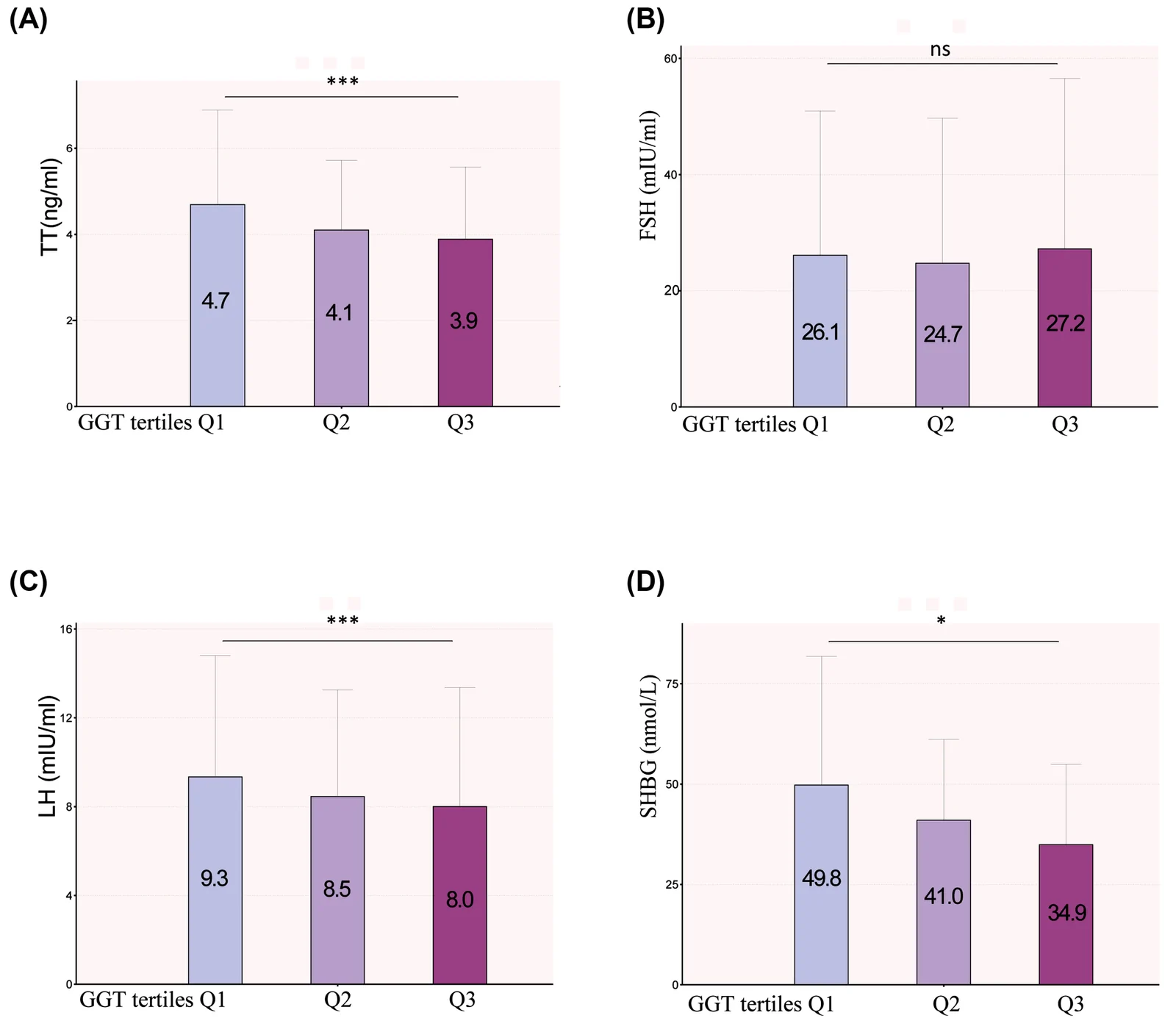
Comparison of male reproductive hormone levels across GGT tertiles. (**A**). TT levels in different GGT tertiles. (**B**). FSH levels in different GGT tertiles. (**C**). LH levels in different GGT tertiles. (**D**). SHBG levels in different GGT tertiles. *** *p* < 0.01, * *p* < 0.05, ^ns^ *p* > 0.05.

Based on the inclusion and exclusion criteria, a total of 971 participants were selected for the final analysis. The baseline characteristics of all participants are shown in Table **1**. The average age was 56.37± 12.49 years. The median GGT level was 27 (range, 19–38) IU/L. The positive correlation was observed with regard to BMI, INS, CP, ALT, TG, TC, LDL, and UA levels among different GGT groups. No significant differences were detected in hypertension, eGFR, and AST, HbA1c, and FPG levels (all *p* values > 0.05). Notably, compared with the other two GGT tertiles, participants in the highest GGT tertile (≥ 33 U/L) exhibited significantly lower levels of TT (*p* < 0.01) (Fig. **2A**), LH (*p* < 0.01) (Fig. **2B**), and SHBG (*p* < 0.05) (Fig. **2C**), while no statistically significant difference was observed in FSH levels (*p* = 0.492) (Fig. **2D**).

### 3.2 Association Between Serum GGT Levels and Serum Male Reproductive Hormone Levels

Table **2** shows the β values and 95% CIs for the association between serum GGT levels and serum male reproductive hormone levels. In the non-adjusted model, the level of TT, LH and SHBG decreased as the tertile of GGT increased (*p* for trend <0.05). However, there were nonsignificant changes in FSH levels. Participants included in the highest GGT tertile (*vs*. the lowest tertile) decreased by 0.81 ng/mL in serum TT level (β -0.81 [95% CI -1.09, -0.52]). After adjustment for age, BMI, CP, ALT, HbA1c, INS, LDL, TG, HDL, UA levels, smoking status and alcohol consumption, the β values were -0.32 (-0.63, -0.01) and -0.44 (-0.78, -0.09) for the second and third GGT tertiles, respectively (*p* for trend =0.013). We converted the GGT levels from a continuous variable to a categorical variable for sensitivity analysis. We found that the *p* for trend for GGT level as a categorical variable was consistent with the result of the fully adjusted model II. In addition, we also found that the trend of the effect size in different GGT groups was non-equidistant. In the unadjusted model, a statistically significant negative association was observed between SHBG and GGT levels (β -14.87 (-26.47, -3.28)). However, this association became non-significant after adjustment for potential confounders. A similar pattern was noted for the relationship between LH and GGT levels, where the initially significant association in unadjusted analysis was attenuated to non-significance in the adjusted model. However, no obvious association or significant P for trend was found for FSH levels.

### 3.3 Threshold Effect Analysis of  GGT  Levels  with Respect to TT

**Fig. (3):**
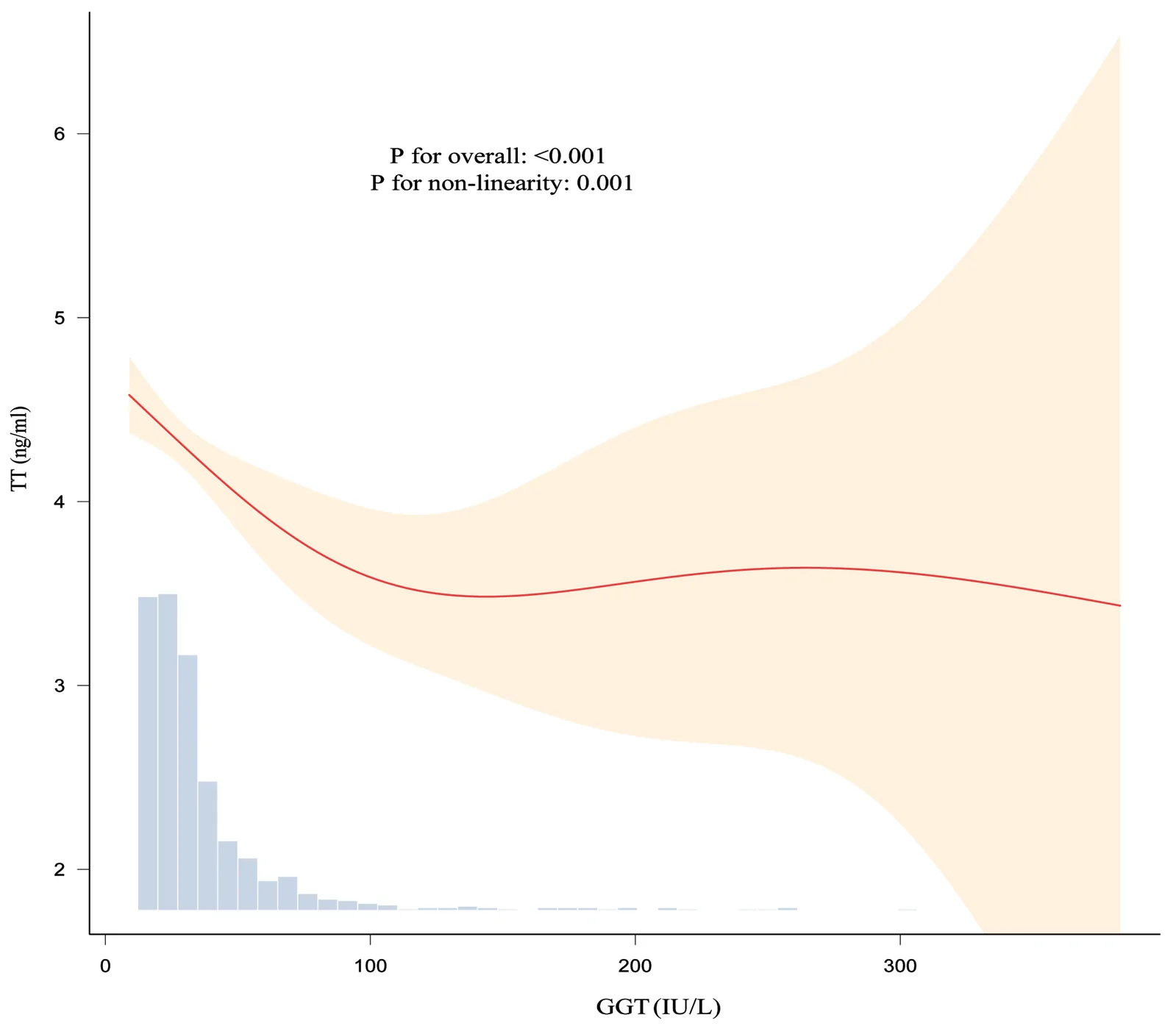
Nonlinear relationship between serum GGT levels and TT levels. Serum TT levels were positively correlated with serum GGT levels, reaching a peak at 118 IU/L.

To assess the dose-response relationship between GGT and TT, we used a smoothing function analysis. After adjusting for age and BMI levels, a nonlinear relationship between serum GGT levels and TT levels was observed (Fig. **3**). Serum TT levels were negatively correlated with serum GGT levels, reaching a peak at 118 IU/L (β -0.013 (-0.019, -0.006), *p* <0.001). However, when the concentration of GGT was higher than 118 IU/L, the β value was -0.003 (-0.016, 0.009), indicating that the TT level did not decrease significantly with an increase in GGT levels (*p* =0.58) (Table **3**). In Fig. (**3**), the red line represents the estimated TT level, and the yellow-shaded areas indicate the point-wise 95% CI.

### 3.4 Subgroup Analyses

Age and hypertension are known confounders of the association between GGT and TT. To understand whether the relationship between serum GGT levels and male reproductive hormone levels is stable in different subgroups, we conducted stratified and interactive analyses (Table **4**). Analysis showed that age and hypertension had no interaction with the association between GGT and TT (*p* >0.05). The associations between GGT and TT were stronger in those in the top two tertiles of GGT levels, but not significant in participants aged < 60 years.

## 4 DISCUSSION

In this cross-sectional study, GGT was found to be negatively correlated with TT, as an independent marker of increased oxidative stress [19], independent of age, BMI, CP, ALT, HbA1c, INS, LDL, TG, HDL, UA levels, smoking status, and alcohol consumption. Furthermore, we revealed a nonlinear relationship between GGT and TT. To our knowledge, our study is the first to evaluate the dose-response relationship between GGT and TT systematically. The level of TT decreased significantly by 0.13 ng/mL for every 10-IU/L increase in GGT levels when the GGT level was <118 IU/L, but the serum TT levels almost leveled off when the GGT level was >118 IU/L, which clarifies previous inconsistent findings on the relationship between GGT and TT in men.

There exists a bidirectional interaction between GGT and TT. First, studies have demonstrated that TT may modulate GGT levels. Preclinical study [20] showed that high- dose testosterone enanthate (80 mg/kg/week) administration in male Wistar rats significantly elevated serum GGT activity (along with ALP) without concurrent increases in AST or ALT levels. While, two discordant studies [21, 22] have demonstrated that adolescent male rats undergoing 12-week moderate-intensity endurance training (EndTr) and receiving weekly intragastric administration (5 doses/week at 2 mg/kg BW) of fluoxymesterone, methylandrostanolone, or stanozolol during the final 8 weeks showed no significant elevations in serum hepatic enzymes (AST, ALT, ALP, or GGT). Complementing the animal evidence, multicenter prospective study of 1933 transgender individuals, including 1044 transgender men who received various testosterone formulations (including gels, esters, and undecanoate), which showed stable GGT levels at both 3- and 12-month follow-ups in both transgender men and transgender women, with an exceptionally low incidence of liver injury (0.1-0.6%) [23]. No significant difference in GGT levels before and after testosterone supplementation was also found in randomized controlled trials of healthy older men [24] and men with late-onset hypogonadism [25]. Membrane-bound GGT plays a crucial role in maintaining cellular redox homeostasis through glutathione metabolism. Serum GGT appears to serve as a nonspecific marker of systemic oxidative stress [26]. Even when testosterone-induced GGT changes occur, they likely reflect a physiological response to oxidative stress rather than pathological liver damage, providing important context for interpreting GGT fluctuations during androgen therapy.

In turn, elevated GGT has been linked to reduced TT. Beyond its presence in the liver and kidneys, GGT is also expressed in various components of the male reproductive system, including Sertoli cells, Leydig cells, and the epithelial linings of the epididymis, seminal vesicles, and vas deferens [27]. Notably, GGT5, primarily found in Leydig cells, has been linked to testosterone deficiency in type 2 diabetic mice, suggesting its role in maintaining the testosterone-producing microenvironment [28]. Potential mechanisms include oxidative damage or dysregulated P38 signaling, which may disrupt extracellular signal-related kinase activity and reduce mitochondrial steroidogenic acute regulatory protein levels. Another proposed pathway involves GGT5-induced HO-1 overexpression, which depletes heme stores and impairs cytochrome P450 enzymes essential for steroidogenesis. *In vitro* studies support that low testosterone [29] and androgen-deprivation therapy can exacerbate oxidative stress [30]. However, the causal link between GGT and TT remains to be confirmed through cohort studies or Mendelian randomization. Additionally, while the correlation between serum and tissue GGT levels is unclear, distinguishing between these may help clarify organ-specific contributions to elevated serum GGT [31].

As a component of the Fatty Liver Index (FLI), GGT is closely tied to nonalcoholic fatty liver disease (NAFLD). Existing research highlights an inverse relationship between NAFLD and testosterone. A meta-analysis [32] involving 2,995 NAFLD patients from 10 published cross-sectional studies revealed that men with moderate-to-severe NAFLD had lower TT than those with mild cases. Similarly, using the 2013–2016 National Health and Nutrition Examination Survey, Donghee *et al.* [33] analyzed data from 8,687 participants (49.5% male) and observed that low testosterone levels correlated with increased NAFLD risk in men, while a higher T/E2 ratio was protective. Our previous work [34] further supports that FLI was the strongest risk factor for low testosterone levels in males across different age groups, irrespective of insulin resistance (IR). Testosterone plays a pivotal role in glucose and lipid metabolism, with its deficiency contributing to IR—a key driver of NAFLD pathogenesis [35]. IR elevates free fatty acids, boosts triglyceride and LDL production, and triggers oxidative stress, accelerating NAFLD progression [36, 37]. Moreover, low TT may promote NAFLD by enhancing lipid synthesis while suppressing fatty acid oxidation, leading to visceral fat accumulation [38, 39]. Future studies should investigate whether GGT and TT serve as mediating factors in NAFLD development.

In addition, we also studied the association between GGT and other sex hormones, including FSH, LH and SHBG in men with diabetes. Our study found a significant association between GGT and SHBG, as well as between GGT and LH, but these associations disappeared after adjusting for age and BMI. No obvious relationships were found with regard to FSH. Only one previous cross-sectional study of 279 patients examined the correlation between GGT and SHBG in men with diabetes and reported that age- and sex-adjusted SHBG concentrations were negatively associated with GGT levels [18]. Generally, patients with peripheral hypogonadism have higher LH levels accompanied by low testosterone levels. High glycemic levels, observed in T2DM patients, diminish the amplitude of the gonadotropin-releasing hormone pulse in men [40, 41], consequently, decreasing LH levels, which may explain our findings regarding the decrease in LH.

The potential limitations of the present study were as follows: (1) it was a cross-sectional study; thus, we could not demonstrate a causal link between GGT and male reproductive hormones; (2) the study sample only comprised Chinese men with diabetes, limiting the generalizability of our findings; (3) measurements of serum estradiol were lacking. And (4) the results were based on a single measurement, the serum T level. Although we attempted to minimize natural diurnal variation in the T level by drawing blood samples between 6:00 and 9:00 AM on the day of screening, the results from a single measurement may not fully represent the real serum T level of the participants.

The present study also had several strengths. First, to our knowledge, this was the only study to evaluate the independent association between GGT and male reproductive hormones, including TT, SHBG, FSH, and LH, in men with type 2 diabetes. Second, our sample size was relatively large compared with those in previous similar studies. Third, we addressed the nonlinearity in the present study and explored this further. We used strict statistical adjustment to minimize the effects of residual confounders. Finally, effect modifier analysis was performed to reach stable conclusions in different subgroups in this study. The findings of this study should be helpful for future research on identifying high-risk populations that require hormonal evaluation and understanding their potential clinical relevance to antioxidant therapy.

## CONCLUSION

This study investigated the independent association between GGT and sex hormone levels in male patients with T2DM. The results demonstrated a significant non-linear correlation between GGT levels and total TT levels. These findings suggest that GGT may serve as a potential biomarker for assessing low testosterone risk in male T2DM patients, support with potential clinical relevance to antioxidant therapy. Subsequent cohort studies could be conducted to validate these findings further.

## Figures and Tables

**Table 1:** Baseline characteristics of participants by categories of serum GGT levels.

**Variable**	**All Participants**	**Serum GGT Tertiles**			***p*-Value**
		**Q1 (≤21U/L)**	**Q2 (22-32U/L)**	**Q3 (≥ 33U/L)**	
Participants (n)	971	323	318	330	-
Age (years)	56.37 ± 12.49	59.93 ± 12.35	56.84 ± 11.66	52.14 ± 12.15	<0.001
Hypertension (%)	503 (51.80%)	164 (50.77%)	168 (52.83%)	171 (51.82%)	0.873
BMI (Kg/m^2^)	26.1 ± 3.4	25.2 ± 3.1	26.3 ± 3.4	26.8 ± 3.6	<0.001
TT (ng/ml)	4.00 (2.99-5.19)	4.38 (3.18-5.70)	3.96 (3.05-5.05)	3.63 (2.69-4.77)	<0.001
FSH (mIU/ml)	12.45 (6.14-42.91)	13.64 (6.48-43.21)	11.97 (6.02-40.07)	10.94 (5.65-45.15)	0.55
LH (mIU/ml)	7.48 (5.34-10.46)	8.27 (5.99-11.18)	7.47 (4.82-10.20)	6.67 (5.13-9.47)	<0.001
SHBG (nmol/L)	35.58 (26.47-52.84)	38.70 (28.42-58.99)	35.58 (25.02-52.59)	31.49 (24.75-41.06)	0.046
HbA1c (%)	8.54 ± 1.86	8.46 ± 1.91	8.58 ± 1.89	8.55 ± 1.71	0.51
INS (µU/ml)	7.28 (4.20-12.75)	5.82 (3.37-9.99)	7.46 (4.64-12.61)	9.14 (5.18-14.28)	<0.001
CP (ng/ml)	2.02 ± 1.25	1.62 ± 1.12	2.01 ± 1.30	2.44 ± 1.20	<0.001
FPG (mmol/L)	8.35 ± 2.93	8.38 ± 3.03	8.38 ± 2.84	8.29 ± 2.95	0.816
eGFR	99.10 ± 24.14	98.79 ± 25.74	98.11 ± 24.31	99.84 ± 22.51	0.751
GGT (U/L)	27.00 (19.00-38.00)	17.00 (14.00-19.00)	27.00 (24.00-29.00)	47.00 (38.00-68.00)	<0.001
ALT (U/L)	19.00 (14.00-28.00)	15.00 (12.00-20.00)	19.00 (14.00-26.00)	26.50 (19.00-40.00)	<0.001
AST (U/L)	19.00 (16.00-24.00)	19.00 (16.00-24.00)	19.00 (16.00-24.00)	19.00 (16.00-24.00)	0.603
LDL (mmol/L)	2.96 ± 0.93	2.78 ± 0.85	2.95 ± 0.92	3.16 ± 0.97	<0.001
TC (mmol/L)	4.74 ± 1.31	4.51 ± 1.21	4.70 ± 1.32	5.02 ± 1.36	<0.001
TG (mmol/L)	1.41 (0.98-2.04)	1.05 (0.80-1.55)	1.40 (0.99-1.94)	1.86 (1.28-2.67)	<0.001
HDL (mmol/L)	1.10 ± 0.29	1.17 ± 0.32	1.08 ± 0.26	1.06 ± 0.29	<0.001
UA (µmol/L)	342.83 ± 91.93	325.54 ± 79.96	347.16 ± 91.72	356.39 ± 100.90	<0.001
Smoking status (%)	-	-	-	-	0.009
never	364 (37.8)	131 (40.7)	121 (38.4)	112 (34.4)	-
ever	235 (24.4)	91 (28.3)	76 (24.1)	68 (20.9)	-
current	364 (37.8)	100 (31.1)	118 (37.5)	146 (44.8)	-
Alcohol consumption (%)	-	-	-	-	< 0.001
never	325 (33.7)	143 (44.4)	105 (33.3)	77 (23.6)	-
ever	166 (17.2)	54 (16.8)	63 (20)	49 (15)	-
current	472 (49.0)	125 (38.8)	147 (46.7)	200 (61.3)	-

**Note:** Data are presented as the mean ± SD, median (*Q*1–*Q*3), or *N *(%).

**Table 2:** Association between serum GGT levels and male reproductive hormone levels.

Variable	**Quintile of GGT**			*p* for Trend
	**Q1 (≤21U/L)**	**Q2 (22-32U/L)**	**Q3 (≥ 33U/L)**	
TT (ng/ml)	-	-	-	-
Non-adjusted	0(ref)	-0.59 (-0.88, -0.30)	-0.81 (-1.09, -0.52)	<0.001
Adjust I	0 (ref)	-0.45 (-0.73, -0.16)	-0.62 (-0.92, -0.32)	<0.001
Adjust II	0 (ref)	-0.32 (-0.63, -0.01)	-0.44 (-0.78, -0.09)	0.013
FSH (mIU/ml)	-	-	-	-
Non-adjusted	0 (ref)	-1.38 (-5.49, 2.72)	1.09 (-2.97, 5.16)	0.593
Adjust I	0 (ref)	-1.44 (-5.68, 2.8)	1.03 (-3.33, 5.39)	0.644
Adjust II	0 (ref)	-1.8 (-6.45, 2.85)	0.68 (-4.57, 5.93)	0.843
LH (mIU/ml)	-	-	-	-
Non-adjusted	0 (ref)	-0.89 (-1.70, -0.08)	-1.34 (-2.14, -0.54)	0.001
Adjust I	0 (ref)	-0.32 (-1.08, 0.44)	0.23 (-0.55, 1.01)	0.565
Adjust II	0 (ref)	-0.27 (-1.12, 0.58)	0.35 (-0.6, 1.31)	0.508
SHBG (nmol/L)	-	-	-	-
Non-adjusted	0 (ref)	-8.79 (-21.18, 3.61)	-14.87 (-26.47, -3.28)	0.013
Adjust I	0 (ref)	-2.47 (-12.95, 8)	-3.36 (-13.5, 6.79)	0.518
Adjust II	0 (ref)	-1.52 (-11.32, 8.29)	-7.69 (-21.13, 5.74)	0.305

**Note:** Data are presented as β values and 95% confidence intervals.

**Table 3:** Threshold effect analysis of GGT levels on the levels of TT.

**Outcome **	**β (95% CI)**	***p*-Value**
One-line linear regression model	-0.01 (-0.01, -0.00)	<0.001
Two-piecewise linear regression model	-	-
GGT<118 U/L	-0.013(-0.019,-0.006)	<0.001
GGT≥ 118 U/L	-0.003 (-0.016, 0.009)	0.58
Log-likelihood ratio test	0.183	-

**Notes:** adjusted for age and BMI.

**Table 4:** Subgroup analyses of the association between serum GGT levels and TT.

**Confounding Factor **	**N**	**Serum GGT Tertiles**			***p* for Trend**	***p* for Interaction**
		**Q1 (≤21U/L)**	**Q2 (22-32U/L)**	**Q3 (≥ 33U/L)**		
Age (years)	-	-	-	-	-	-
<60	563	0(ref)	-0.25 (-0.64, 0.13)	-0.36 (-0.75, 0.02)	0.0726	0.9408
≥ 60	408	0(ref)	-0.09 (-0.57, 0.38)	-0.13 (-0.73, 0.47)	0.6424	-
Hypertension (%)	-	-	-	-	-	-
No	468	0(ref)	0.01 (-0.43, 0.44)	-0.13 (-0.61, 0.34)	0.5861	0.6503
Yes	503	0(ref)	-0.36 (-0.78, 0.05)	-0.36 (-0.82, 0.10)	0.1155	-

## Data Availability

The data supporting the findings of this study will be available from the corresponding author upon reasonable request.

## LIST OF ABBREVIATIONS

GGT: Gamma-glutamyl Transferase
T2DM: Type 2 Diabetes Mellitus
DM: Diabetes Mellitus
TT: Total Testosterone
FSH: Follicle-stimulating Hormone
LH: Luteinizing Hormone
SHBG: Sex Hormone-binding Globulin
DHEAS: Dehydroepiandrosterone Sulfate
BMI: Body Mass Index
HbA1c: Glycated Hemoglobin
INS: Insulin
CP: C-peptide
eGFR: Estimated Glomerular Filtration Rate
ALT: Alanine Aminotransferase
AST: Aspartate Aminotransferase
TG: Triglyceride
TC: Total Cholesterol
HDL: High-density Lipoprotein Cholesterol
LDL: Low-density Lipoprotein Cholesterol
UA: Uric Acid
FPG: Fasting Plasma Glucose
SD: Standard Deviation
FLI: Fatty Liver Index
NAFLD: Nonalcoholic Fatty Liver Disease
IR: Insulin Resistance

## STANDARDS OF REPORTING

STROBE guidelines were followed.
